# Sample-constrained partial identification with application to selection bias

**DOI:** 10.1093/biomet/asac042

**Published:** 2022-07-25

**Authors:** Matthew J Tudball, Rachael A Hughes, Kate Tilling, Jack Bowden, Qingyuan Zhao

**Affiliations:** MRC Integrative Epidemiology Unit, University of Bristol, Oakfield Grove, Bristol, BS8 2BN, U.K; MRC Integrative Epidemiology Unit, University of Bristol, Oakfield Grove, Bristol, BS8 2BN, U.K; MRC Integrative Epidemiology Unit, University of Bristol, Oakfield Grove, Bristol, BS8 2BN, U.K; College of Medicine and Health, University of Exeter, Heavitree Road, Exeter, EX1 2LU, U.K; Department of Pure Mathematics and Mathematical Statistics, University of Cambridge, Wilberforce Road, Cambridge CB3 0WB, U.K

**Keywords:** Auxiliary information, Constraint, Partial identification, Selection bias, Sensitivity analysisa

## Abstract

Many partial identification problems can be characterized by the optimal value of a function over a set where both the function and set need to be estimated by empirical data. Despite some progress for convex problems, statistical inference in this general setting remains to be developed. To address this, we derive an asymptotically valid confidence interval for the optimal value through an appropriate relaxation of the estimated set. We then apply this general result to the problem of selection bias in population-based cohort studies. We show that existing sensitivity analyses, which are often conservative and difficult to implement, can be formulated in our framework and made significantly more informative via auxiliary information on the population. We conduct a simulation study to evaluate the finite sample performance of our inference procedure, and conclude with a substantive motivating example on the causal effect of education on income in the highly selected UK Biobank cohort. We demonstrate that our method can produce informative bounds using plausible population-level auxiliary constraints. We implement this method in the $\texttt{R}$ package $\texttt{selectioninterval}$.

## 1. Introduction

### 1.1. General problem

Partial identification problems arise when the observable data are only sufficient to identify a set or interval in which a parameter of interest is contained. A classical example from Manski (2003) is the missing data problem, where $Y$ is a discrete random variable and $S$ is a binary random variable indicating whether $Y$ is observed ($S = 1$) or not ($S = 0$). The distribution of $Y$ can be decomposed into

$$
\begin{equation*}
{\rm{pr}}(Y = y) = {\rm{pr}}(Y = y \mid S = 1) {\rm{pr}}(S = 1) + {\rm{pr}}(Y = y \mid S = 0) {\rm{pr}}(S = 0)
\end{equation*}$$

for any $y$ in the support of $Y$. Given that ${\rm{pr}}(Y = y \mid S = 0)$ is unobserved, the smallest value that ${\rm{pr}}(Y = y)$ could take is ${\rm{pr}}(Y = y \mid S = 1) {\rm{pr}}(S = 1)$ and the largest value is ${\rm{pr}}(Y = y \mid S = 1) {\rm{pr}}(S = 1) + {\rm{pr}}(S = 0)$. Therefore, although ${\rm{pr}}(Y = y)$ itself cannot be point identified, it can be partially identified via the interval corresponding to the smallest and largest possible values.

Many partial identification problems can be formulated as the optimal value of a population objective function, which we write as

(1) \begin{equation*}\label{eqn:inference-target} \nu = \inf \{Q(\theta) \colon \theta \in \Theta \}, \end{equation*}

where $Q \colon \mathbb{R}^p \to \mathbb{R}$ and $\Theta \subseteq \mathbb{R}^p$. In the missing data example, $Q(\theta) = {\rm{pr}}(Y = y \mid S = 1) {\rm{pr}}(S = 1) + \theta {\rm{pr}}(S = 0)$ and $\Theta = [0, 1]$.

The field of stochastic optimization also considers problems of the form in (1) and has built a large literature on estimation of, and inference to, $\nu$ when a sample analogue $Q_n$ is observed instead of $Q$, where $n$ denotes the sample size. We demonstrate that framing the partial identification problem as a stochastic optimization problem will allow us to draw upon these existing results.

Specifically, in this article we are concerned with the difficult setting where $\Theta$ must also be estimated empirically. We consider a setting where $\Theta$ is characterized by inequality constraints of the form $\Theta = \{\theta \colon h_j(\theta) \leqslant 0, j = 1, \ldots, J \}$, where we may only observe corresponding estimators $h_{nj}(\theta)$. Within this setting, our goal is to find a lower confidence bound $C_n$ for any $0 < \alpha < 1$ such that

(2) \begin{equation*}\label{eqn:confidence-bound} \lim_{n \to \infty} {\rm{pr}}(C_n \leqslant \nu) \geqslant 1 - \alpha, \end{equation*}

which will suffice to provide useful statistical inference in a wide set of applications.

### 1.2. Motivating application

Our investigation is motivated by an applied question: how will selection bias affect the conclusions of population-based cohort studies? Many statistical analyses begin by selecting a study sample from some population of interest. When the sample is drawn nonrandomly, then valid inference for the population is no longer guaranteed (Bareinboim et al., 2014). Inverse probability weighting could be used to correct this selection bias (Horvitz & Thompson, 1952; Stuart et al., 2011), but data on nonselected observations may be limited or unavailable altogether, such that the weights cannot be estimated. In such settings, there exist approaches to assess the sensitivity of estimates to a range of plausible inverse probability weights (Aronow & Lee, 2013; Thompson & Arah, 2014). However, these approaches could be made more informative via a principled procedure for conducting statistical inference and the inclusion of relevant auxiliary information about the population. We demonstrate that such improvements can be made by casting these sensitivity analyses within the general framework described in § 1.1.

We are specifically motivated by studies conducted in the UK Biobank, which is a large population-based cohort study widely analysed by health researchers. Studies of this cohort are potentially biased since recruited participants are known to differ systematically from the rest of the U.K. population on measures such as education, health status, age and geographical location (Fry et al., 2017; Hughes et al., 2019).

### 1.3. Existing literature

Statistical inference procedures have been developed for some special cases of our general problem in (2). An area of particular focus is the so-called ‘sample average approximation’ (Shapiro et al., 2009). In this case, $Q$ is the expected value $Q(\theta) = E\{f(\theta, X)\}$ of some function $f$ and $Q_n$ is a sample average $Q_n(\theta) = n^{-1} \sum_{i=1}^n f(\theta, X_i)$, where $X$ is some random variable and $X_1, \ldots, X_n$ are independent draws of $X$.

Statistical inference in the presence of $\Theta_n$ has been developed for convex sample average approximations, such that $f$ is convex in $\theta$ and $\Theta = \{ \theta \colon h_j(\theta) \leqslant 0, j = 1, \ldots, J \}$, where $h_j(\theta) = E\{g_j(\theta, X)\}$ and $g_j(\theta, X)$ is convex in $\theta$ for all $j$. Shapiro (1991) showed that the plug-in estimator

(3) \begin{equation*} \label{eqn:plug-in-estimator} \nu_n^p = \inf \{ Q_n(\theta) \colon \theta \in \Theta_n \} \end{equation*}

satisfies a central limit theorem under these convexity assumptions, and some additional regularity conditions, where $\Theta_n = \{ \theta \colon \sum_{i=1}^n g_j(\theta, X_i) \leqslant 0, j = 1, \ldots, J \}$.

Moving away from convex problems, Wang & Ahmed (2008) considered the special case of minimizing a known function $Q$ subject to a single expected value constraint $\Theta = \{ \theta \colon E\{g(\theta, X)\} \leqslant 0 \}$. They proposed an approach for calculating a sample size $n$ so that $\Theta_n$ is feasible to some small relaxation of the true problem with high probability.

Our work also overlaps with the partial identification literature in econometrics, much of which considers inference for identified sets characterized by conditional or unconditional moment inequalities, commonly interpreted as the set of minimizers of some criterion function (Chernozhukov et al., 2007; Andrews & Soares, 2010; Andrews & Shi, 2013). A related literature provides inference for parameters lying within partially identified sets, as opposed to inference for the set itself (Imbens & Manski, 2004; Stoye, 2009). For a more comprehensive review of the partial identification literature, see Molinari (2020).

## 2. Confidence intervals for sample-constrained partial identification

### 2.1. Confidence intervals under known constraints

In this section, we briefly summarize existing results on statistical inference for stochastic optimization when the set $\Theta$ is observed, which forms the basis of our generalization to situations where an estimate $\Theta_n$ of $\Theta$ is observed instead. Suppose that the parameter space is defined by a set of inequality constraints

$$
\begin{equation*}
\Theta = \{ \theta \colon h_j(\theta) \leqslant 0, \, j = 1, \ldots, J \},
\end{equation*}$$

where an equality constraint for some $h_j(\theta)$ can be introduced by taking the inequality constraints of both $h_j(\theta)$ and $-h_j(\theta)$. Recall that our goal is to provide inference about the infimum $\nu = \inf \{Q(\theta) \colon \theta \in \Theta \}$.

Much of the literature in stochastic optimization is centred on the statistical properties of the estimator

(4) \begin{equation*}\label{eqn:sample-estimator} \nu_n = \inf \{Q_n(\theta) \colon \theta \in \Theta \}. \end{equation*}

Consistency of optimal values and optimal solutions to such stochastic optimization problems is typically achieved by imposing uniform convergence of $Q_n(\theta)$ to $Q(\theta)$. First-order asymptotic properties are obtained via the functional delta method. The key conditions are that the infimum, viewed as a function of $Q$, satisfies some notion of differentiability at $Q$ and that $n^{-1/2}(Q - Q_n)$ converges to a Gaussian process; see Shapiro (1991) for further details.

To make the previous discussion more concrete, consider the following four assumptions commonly placed on the stochastic optimization problem described above.

*Assumption* 1. The set of solutions to (1) is a singleton $\{\theta \in \Theta \colon Q(\theta) = \nu \} = \{\vartheta\}$.

*Assumption* 2. Let $B \subseteq \mathbb{R}^p$ denote a compact set and $C(B)$ denote the space of continuous functions on domain $B$. Then $\Theta \subseteq B$, $Q \in C(B)$ and $Q_n \in C(B)$ with probability $1$.

*Assumption* 3. It holds that $Q_n(\theta)$ converges to $Q(\theta)$ with probability $1$ as $n \to \infty$ uniformly on $B$.

*Assumption* 4. As $n \to \infty$, the sequence $V_n(\theta) = n^{1/2}\{Q(\theta) - Q_n(\theta)\}$ converges in distribution to a random element $V(\theta) \in C(B)$, where $V(\theta)$ is a Gaussian process with mean 0 and variance $\sigma^2(\theta) \in C(B)$.

These assumptions are jointly sufficient to achieve consistency and asymptotic normality of $\nu_n$, which we state formally in the following two propositions.

Proposition 1. *Let $\vartheta_n \in \text{arg}\,\text{min} \{ Q_n(\theta) \colon \theta \in \Theta \}$ be a sample solution, and let $\nu_n$ be defined as in* (4)*. Under Assumptions* 1, 2 *and* 4*, $\nu_n \to \nu$ and $\vartheta_n \to \vartheta$ with probability $1$.*

Proposition 1 is identical to Theorem 5.3 of Shapiro et al. (2009) under the condition that $\vartheta$ is unique.

Proposition 2. *Under Assumptions* 1, 2 *and* 4,

$$
\begin{equation*}
n^{1/2} (\nu_n - \nu) \to \mathcal{N}\{0, \sigma^2(\vartheta)\}
\end{equation*}$$

*in distribution, where $\sigma^2(\vartheta)$ is the asymptotic variance of $\nu_n$ defined in Assumption* 4.

Proposition 2 is an immediate consequence of Theorem 3.2 of Shapiro (1991). Although we do not restate the proof here, the intuition is that Assumptions 1 and 2 allow a notion of differentiability of the infimum, and Assumption 4 provides weak convergence of $n^{1/2} (Q - Q_n)$ to a Gaussian process, thus providing the conditions needed for an application of the delta method.

To use Proposition 2 to construct a valid confidence interval, we must take into consideration that both $\sigma^2$ and $\vartheta$ are unknown. To this end, we state an additional assumption followed by a proposition.

*Assumption* 5. There exists a uniformly strongly consistent estimator $\sigma_n^2(\theta) \in C(B)$ for $\sigma^2(\theta)$ such that $\sup_{\theta \in \Theta} |\sigma_n^2(\theta) - \sigma^2(\theta)| \to 0$ with probability $1$.

Proposition 3. *Under Assumptions* 1, 2, 3 *and* 5, *$\sigma^2_n(\vartheta_n) \to \sigma^2(\vartheta)$ with probability $1$.*

Assumption 5 applies uniform convergence to an estimator for the asymptotic variance of $Q_n(\theta)$. This strong notion of convergence for $\sigma_n^2(\vartheta_n)$ allows us to construct a confidence bound of the form

(5) \begin{equation*} \label{eqn:pop-confidence-bound} C_n = \nu_n - Z_{\alpha} \sigma_n(\vartheta_n) n^{-1/2}, \end{equation*}

where $Z_{\alpha}$ is the upper $\alpha$-quantile of the standard normal distribution. This choice of $C_n$ has asymptotically exact nominal coverage $1-\alpha$ by Proposition 2, Proposition 3 and Slutsky’s theorem.

### 2.2. Confidence intervals under sample constraints

We now consider the more difficult setting where the constraint functions $h_j(\theta)$ need to be estimated as well. We instead observe an estimator $\Theta_n = \{ \theta \colon h_{nj}(\theta) \leqslant 0, j = 1, \ldots, J \}$ comprised of estimators of the constraint functions $h_{nj}(\theta)$. We discuss what properties $\Theta_n$ must have to allow valid statistical inference for $\nu$.

It is tempting to follow the approach of the previous section and construct a plug-in estimator for $\nu$ by simply replacing $Q$ with $Q_n$, and $\Theta$ with $\Theta_n$, and finding the corresponding infimum. This is the approach taken by Shapiro et al. (2009), given by $\nu_n^p$ in (3). A problem with this approach is that it is possible that $\Theta \cap \Theta_n = 0$ with probability $1$ even as $n$ becomes large. This means that the true solution $\vartheta$ will almost never lie inside $\Theta_n$, prohibiting the construction of a valid confidence interval for $\nu$, as illustrated by the contrived example below.

*Example* 1. Consider a problem of the form $Q(\theta) = \theta^2 + E(X)$ and $\Theta = \{\theta \colon \theta = E(X) \}$, where $X \sim \mathcal{N}(1,1)$ is a normally distributed random variable. The plug-in estimators are $Q_n(\theta) = \theta^2 + \bar{X}_n$ and $\Theta_n = \{\theta \colon \theta = \bar{X}_n \}$, where $\bar{X}_n$ is the mean of $n$ independent and identically distributed draws of $X$. It follows that $\nu = 2$ and $\nu_n^p = \bar{X}_n^2 + \bar{X}_n $, where $\nu_n^p$ is the plug-in estimator in (3). The asymptotic variance of $Q_n(\theta)$ is $\sigma^2(\theta) = 1$, which we assume is known. The confidence bound in (2) is $C_n = \bar{X}_n^2 + \bar{X}_n - Z_{\alpha} n^{-1/2}$. A simple Monte Carlo simulation demonstrates that the corresponding 95$\%$ confidence interval for $n = 100$ exhibits subnominal coverage of around 70$\%$.

Existing approaches in stochastic optimization address the problem in Example 1 by restricting to sample average approximations, and imposing convexity of both $Q$ and $h$. To allow inference for a broader class of problems, we propose an intuitive but conservative approach that replaces $\Theta_n$ with an appropriate relaxation. In particular, we propose to use the relaxed set

(6) \begin{equation*} \label{eqn:relaxed-constraint} \Theta_n^r = \{ \theta \colon h_{nj}(\theta) \leqslant \epsilon_{nj}(\theta), \, j = 1, \ldots, J\}, \end{equation*}

where $\epsilon_n(\theta) = \{\epsilon_{n1}(\theta), \ldots, \epsilon_{nJ}(\theta)\}^{ \mathrm{\scriptscriptstyle T} }$ is some $J$-dimensional sequence such that $\epsilon_{nj}(\theta) \geqslant 0$ for all $\theta \in B$, chosen so that

(7) \begin{equation*} \label{eqn:constraint-coverage} \lim_{n \to \infty} {\rm{pr}}(\Theta \subseteq \Theta_n^r) \geqslant 1 - \alpha_1 \end{equation*}

for some $0 < \alpha_1 < 1$. The exact forms of $\Theta_n^r$ and $\epsilon_n(\theta)$ are not crucial for our main results, provided (7) holds, which we discuss in more detail toward the end of this section.

Our proposed confidence bound is of the form $C_n(\theta) = Q_n(\theta) - Z_{\alpha_2} \sigma_n(\theta) n^{-1/2}$ for some $0 < \alpha_2 < 1$, where $Z_{\alpha_2}$ is the upper $\alpha_2$-quantile of the standard normal distribution. We need to select a $\theta$ so that (2) is satisfied. This is accomplished by finding the optimal value and solution over the relaxed constraint set,

(8) \begin{equation*} \label{eqn:relaxed-optimum} \nu_n^r = \inf \{Q_n(\theta) \colon \theta \in \Theta_n^r \} \quad\text{and}\quad \vartheta_n^r \in \text{arg}\,\text{min} \{Q_n(\theta) \colon \theta \in \Theta_n^r \}, \end{equation*}

and constructing a confidence bound of the form

(9) \begin{equation*} \label{eqn:relaxed-confidence-bound} C_n = C_n(\vartheta_n^r) = \nu_n^r - Z_{\alpha_2} \sigma_n(\vartheta_n^r) n^{-1/2}. \end{equation*}

We now need to demonstrate that $C_n$ covers $\nu$ with known probability in the limit. To this end, we need an additional technical assumption to hold.

*Assumption* 6. Let $\zeta_n^r \in \text{arg}\,\text{min} \{C_n(\theta) \colon \theta \in \Theta_n^r \}$ be the optimal solution of $C_n(\theta)$ over $\Theta_n^r$. Then $\vert \zeta_n^r - \vartheta_n^r \vert$ converges to $0$ in probability.

Assumption 6 is imposed so that two important quantities become asymptotically close. The first quantity is $C_n(\zeta_n^r)$, which is the infimum over all confidence bounds in $\Theta_n^r$. This confidence bound is important because it provides a lower bound for other quantities with known coverage probabilities, which is a fact we utilize in our main result in Theorem 1. The second quantity is $C_n(\vartheta_n^r)$, which is our main confidence bound proposed in (9).

We argue that Assumption 6 is reasonable in the sense that $\zeta_n^r$ and $\vartheta_n^r$ are solutions over two objective functions that converge uniformly to the same limit. To make this intuition more concrete, we provide some sufficient conditions for Assumption 6 in the Supplementary Material. Essentially, if Assumption 3 is satisfied, and $h_{nj}(\theta)$ and $\epsilon_{nj}(\theta)$ converge to $h_j(\theta)$ and 0 for all $j = 1, \ldots, J$ uniformly on $B$ with probability $1$, then we can show that both $\vartheta_n^r$ and $\zeta_n^r$ converge to $\vartheta$ with probability $1$.

We claim that $C_n$ provides an asymptotically valid lower confidence bound.

Theorem 1. *Suppose that we select a relaxed constraint set $\Theta_n^r$ as in* (6) *and significance level $0 < \alpha_1 < 1$ such that $\lim_{n \to \infty} {\rm{pr}}(\Theta \subseteq \Theta_n^r) \geqslant 1 - \alpha_1$. Suppose that we also select a significance level $0 < \alpha_2 < 1$. Then, under Assumptions* 1–6,

$$
\begin{equation*}\lim_{n \to \infty} {\rm{pr}}( C_n \leqslant \nu) \geqslant 1 - \alpha_1 - \alpha_2.\end{equation*}$$

Here we outline the key steps in the proof. We begin by defining a deterministic sequence $\delta_n = Z_{\alpha_2} \epsilon n^{-1/2}$, where $\epsilon > 0$ is some small constant. We then show that ${\rm{pr}}( C_n \leqslant \nu)$ is bounded from below by the sum of two quantities: ${\rm{pr}}\{C_n(\zeta_n^r) \leqslant \nu - \delta_n\}$ and ${\rm{pr}} \{\vert \sigma_n(\zeta_n^r) - \sigma_n(\vartheta_n^r) \vert \leqslant \epsilon \} - 1$. The second quantity converges to $0$ under Assumption 6. The remainder of the proof follows a similar argument to the main lemma of Berger & Boos (1994). Whenever $\Theta \subseteq \Theta_n^r$, we know that $C_n(\zeta_n^r)$, which is the infimum over all confidence bounds in $\Theta_n^r$, will cover $\nu$ at least as often as $C_n(\vartheta_n)$, which is confidence bound (5). Therefore, ${\rm{pr}}\{C_n(\zeta_n^r) \leqslant \nu, \Theta \subseteq \Theta_n^r\} \geqslant {\rm{pr}}\{C_n(\vartheta_n) \leqslant \nu, \Theta \subseteq \Theta_n^r\}$. We also know that ${\rm{pr}}\{C_n(\vartheta_n) \leqslant \nu, \Theta \subseteq \Theta_n^r\} = {\rm{pr}}\{C_n(\vartheta_n) \leqslant \nu\} - {\rm{pr}}\{C_n(\vartheta_n) \leqslant \nu, \Theta \not\subseteq \Theta_n^r\}$ by the law of total probability. In the limit, the first probability on the right-hand side is equal to $1 - \alpha_2$ by Proposition 2 and the second probability is at most $\alpha_1$ by assumption. This allows us to arrive at our main result.

The proof sketch also provides some insight into why the naive plug-in estimator $\nu_n^p$ defined in (3) may fail to yield a valid confidence interval. A crucial quantity is ${\rm{pr}}(\Theta \subseteq \Theta_n^r)$, which is known under an appropriate choice of $\epsilon_n(\theta)$. The corresponding quantity for the plug-in estimator is ${\rm{pr}}(\Theta \subseteq \Theta_n)$, which could be arbitrarily small. In Example 1, this probability is zero.

It remains to discuss how to construct a relaxed set $\Theta_n^r$. Whenever $\Theta$ can be characterized by a set of moment inequalities, such that $h_j(\theta) = E\{m_j(\theta)\}$, the moment inequalities literature summarized in § 1.3 could be used to construct $\Theta_n^r$. A more conservative relaxed set could be constructed via an application of the intersection bound. Suppose that the following assumption holds on the constraint functions.

*Assumption* 7. For all $\theta \in \Theta$ and $j = 1, \ldots, J$, $n^{1/2} \{h_{nj}(\theta) - h_j(\theta) \} \to \mathcal{N}\{0, \sigma_j^2(\theta) \}$ in distribution and $\sigma_{nj}^2(\theta)$ is a consistent estimator for $\sigma_j^2(\theta)$.

This fairly weak assumption means that $h_{nj}(\theta)$ is pointwise asymptotically normally distributed and that there is a consistent estimator for the variance. This assumption allows us to select

$$
\begin{equation*}
\epsilon_n(\theta) = Z_{\alpha_{1j}} \sigma_{nj}(\theta) n^{-1/2},
\end{equation*}$$

where $\alpha_1 = \alpha_{11} + \alpha_{12} + \cdots + \alpha_{1J}$. It is straightforward to show that this choice of $\epsilon_n(\theta)$ satisfies (7). We could shrink the size of $\Theta_n^r$ by assuming that $h_n(\theta) = \{h_{1,n}(\theta), \ldots, h_{nj}(\theta)\}^{ \mathrm{\scriptscriptstyle T} }$ converges pointwise to a multivariate Gaussian with covariance matrix $\Sigma$ and consistent estimator $\Sigma_n$. This would allow us to construct $\Theta_n^r$ as an ellipsoid confidence region.

*Remark* 1. It remains to discuss how one would select $\alpha_1$ and $\alpha_2$. As a rule of thumb, we typically choose the midpoint $\alpha_1 = \alpha_2 = \alpha/2$. It is tempting to select $\alpha = \alpha_1 + \alpha_2$ and choose $C_n$ as the largest confidence bound over all $\alpha_1$ and $\alpha_2$ satisfying this equality. This would mean that $\alpha_1$ and $\alpha_2$ are sample-dependent quantities and so Theorem 1 will not directly apply. However, we can reason heuristically that the best choice of $\alpha_1$ and $\alpha_2$ should lie at an interior point $\alpha_1 > 0$ and $\alpha_2 > 0$. For a fixed sample, as $\alpha_1 \to 0$ and $\alpha_2 \to \alpha$, $\Theta_n^r \to B$, and thus $C_n$ approaches the $100(1-\alpha)\%$ confidence interval over the unconstrained problem. As $\alpha_1 \to \alpha$ and $\alpha_2 \to 0$, $C_n \to -\infty$, and thus the confidence interval becomes arbitrarily wide.

*Remark* 2. So far, we have focused on inference for the infimum; however, partial identification problems are often characterized by an identified set of the form $I = [\nu^l, \nu^u]$, where $\nu^l = \inf \{Q(\theta) \colon \theta \in \Theta \}$ and $\nu^u = \sup \{Q(\theta) \colon \theta \in \Theta \}$ (Imbens & Manski, 2004; Chernozhukov et al., 2013). Suppose that $\Theta_n^r$ is chosen so that ${\rm{pr}}(\Theta \subseteq \Theta_n^r) \geqslant 1 - \alpha_1/2$. Moreover, let $\nu_n^{r,l} = \inf\{Q_n(\theta) \colon \theta \in \Theta_n^r\}$ and $\nu_n^{r,u} = \sup\{Q_n(\theta) \colon \theta \in \Theta_n^r\}$ denote the optimal values, and let $\vartheta_n^{r,l}$ and $\vartheta_n^{r,u}$ denote the corresponding optimal solutions. The estimated interval can be written as $[\nu_n^{r,l}, \nu_n^{r,u}]$, and we can construct a confidence interval by combining the lower confidence bound for $\nu^l$ and the upper confidence bound for $\nu^u$, so that

$$
[\nu_n^{r,l} - Z_{\alpha_2/2} \sigma_n(\vartheta_n^{r,l}) n^{-1/2}, \nu_n^{r,u} + Z_{\alpha_2/2} \sigma_n(\vartheta_n^{r,u}) n^{-1/2}]
$$

will cover $I$ with probability at least $1-\alpha_1-\alpha_2$. This is the two-sided analogue of the one-sided confidence interval proposed in (9) and Theorem 1.

## 3. Sensitivity analysis via a logistic model

### 3.1. Set-up

We now return to the motivating example of selection bias in population-based cohort studies briefly described in § 1.2. Specifically, we generalize the sensitivity analysis proposed by Thompson & Arah (2014), who defined a logistic model for the probability of sample selection and proposed to select parameters based on domain knowledge, or enumerate a large number of possible parameters. This approach is challenging to implement in the presence of complicated selection mechanisms with many parameters. Plausible sets of parameters that introduce bias in estimates of interest may be overlooked. Therefore, we begin by framing Thompson & Arah (2014) as an optimization problem over a space of plausible parameters, and describe how relevant auxiliary information could be introduced to further restrict the parameter space and provide more informative bounds, such as survey response rates, population means and negative controls. An additional sensitivity analysis, Aronow & Lee (2013), is generalized in the Supplementary Material.

Consider an independent and identically distributed draw of size $N$ from an infinite population. For concreteness, we can think of this finite draw as the set of individuals who are eligible to enter the sample. Let $S_i \in \{0, 1\}$ be a selection indicator for whether individual $i$ enrols in the sample, where $S_i = 1$ indicates sample participation, and let the observed sample size be denoted by $n = \sum_{i=1}^N S_i$. For notational convenience, we assume that $S_1 = \cdots = S_n = 1$ and $S_{n+1} = \cdots = S_N = 0$.

Within the observed sample, we observe a vector of variables related to sample selection $W_i \in \mathbb{R}^K$. As in Thompson & Arah (2014), we assume that the probability of sample selection admits a logistic form,

(10) \begin{equation*} \label{eqn:logit} e(W_i; \theta) = {\rm{pr}}(S_i = 1 \mid W_i) = \frac{\exp(\theta_0 + \theta_1 W_{i1} + \cdots + \theta_k W_{iK})}{1 + \exp(\theta_0 + \theta_1 W_{i1} + \cdots + \theta_k W_{iK})}, \end{equation*}

where $\theta = (\theta_0, \theta_1, \theta_2, \ldots, \theta_K)^{ \mathrm{\scriptscriptstyle T} }$. We further assume that the sample is generated by some true selection probabilities $e(W_i; \theta^*)$ parameterized by $\theta^*$.

For illustration, suppose that our object of interest is the population mean of a random variable $X_i$. We can write the population mean, and corresponding sample mean, in terms of $\theta^*$ as

(11) \begin{equation*}\label{eqn:population-mean} \beta(\theta^*) = E(X_i) = \frac{E\{X_i/e(W_i; \theta^*) \mid S_i = 1\}}{E\{1/e(W_i; \theta^*) \mid S_i = 1\}}, \qquad \beta_n(\theta^*) = \frac{ \sum_{i=1}^n X_i/e(W_i; \theta^*)}{\sum_{i=1}^n 1/e(W_i; \theta^*)}. \end{equation*}

The expression in (11) relies on $X_i \perp S_i \mid W_i$, which we assume throughout.

Since we only observe $X_i$ when $S_i = 1$, the true parameter $\theta^*$ cannot be estimated. Thompson & Arah (2014) proposed to consider a space of plausible values for $\theta^*$, and identified a worst-case lower bound and worst-case upper bound for $\beta_n(\theta^*)$. Inference to $\beta(\theta^*)$ itself was not considered. Formally, we select a parameter space $\Theta$ in which we are confident that $\theta^*$ resides. We then take the infimum and supremum of $\beta_n(\theta)$ over the space $\Theta$.

### 3.2. Sensitivity parameters

Since we have assumed a logistic form for the selection probabilities (10), we can select sensitivity parameters that have a natural interpretation in terms of odds ratios.

Without loss of generality, suppose that each $W_{ik}$ has mean zero and standard deviation one within the sample. We can then choose a parameter $\Lambda_1 \geqslant 1$ such that

(12) \begin{equation*} \label{eqn:lambda1} \Lambda_1^{-1} \leqslant \exp(\theta_k) \leqslant \Lambda_1\quad (k = 1, \ldots, K). \end{equation*}

We can interpret $\Lambda_1$ as the change in the conditional odds of sample selection from a one standard deviation increase in $W_{ik}$, holding all else fixed. When $\Lambda_1 = 1$, sample selection is completely random. Of course, we could select sensitivity parameters $\Lambda_{1k}$ on a variable-by-variable basis for $k = 1, \ldots, K$, although choosing a single $\Lambda_1 = \max_k \Lambda_{1k}$ simplifies the interpretation of the sensitivity analysis.

The intercept term $\theta_0$ also needs to be bounded. We can choose two parameters $\Lambda_0^l, \Lambda_0^u \in (0, 1)$ such that

(13) \begin{equation*} \label{eqn:lambda0} \Lambda_0^l \leqslant \exp(\theta_0) \leqslant \Lambda_0^u, \end{equation*}

which can be interpreted as the odds of sample selection among those for whom $W_{ik} = 0$ for all $k$.

Rearranging (12) and (13) shows that the sensitivity parameters $(\Lambda_0^l, \Lambda_0^u, \Lambda_1)$ characterize a compact subset of $\mathbb{R}^{K+1}$:

(14) \begin{equation*} \label{eqn:example-theta} \theta \in \Theta = [\log(\Lambda_0^l), \log(\Lambda_0^u)] \times [\log(1/\Lambda_1), \log(\Lambda_1)]^K. \end{equation*}

From here, the estimand and estimator can be respectively defined for the worst-case lower bound of $\beta(\theta)$ as

$$
\nu = \inf \{ \beta(\theta) \colon \theta \in \Theta \}, \qquad \nu_n = \inf \{ \beta_n(\theta) \colon \theta \in \Theta \}.
$$

We could of course estimate the worst-case upper bound for $\beta(\theta)$ by taking the supremum of $\beta_n(\theta)$ over $\Theta$; see Remark 2. Naturally, we can also consider estimators other than sample means, such as ordinary least squares or two-stage least squares.

### 3.3. Auxiliary information constraints

We now introduce several common examples, where there may be discordance between known population quantities and quantities implied by the inverse probability weights. In general, provided we can formulate the constraints as a statistical test with a known null distribution, they can be placed within our framework.

*Example* 2. Suppose that we know the response rate for a survey-based sample $r = E\{e(W_i; \theta^*)\}$. It is straightforward to show that $E\{1/e(W_i; \theta^*) \mid S_i = 1\} = 1/r$. This means that the within-sample expectation of the true inverse selection probabilities is equal to the inverse response rate. We therefore only want to consider parameters $\theta$ that imply this inverse response rate. The corresponding constraint can be written as

$$
\begin{equation*}
h_{nj}(\theta) = \frac{1}{n} \sum_{i=1}^n \{1/e(W_i; \theta) - 1/r\} \leqslant Z_{\alpha_{1j}/2} \sigma_{nj}(\theta) / n^{1/2},
\end{equation*}$$

where $\sigma_{nj}(\theta)$ is the sample standard deviation of $1/e(W_i; \theta)$.

*Example* 3. Suppose that we know the population mean $E(W_{ik})$ of some $W_{ik} \in W_i$. The inverse-probability-weighted sample mean of $W_{ik}$ should therefore equal this mean in expectation, since

$$
\frac{E\{W_{ik}/e(W_i; \theta^*) \mid S_i = 1\}}{E\{1/e(W_i; \theta^*) \mid S_i = 1\}} = E(W_{ik}).
$$

This is conceptually similar to the raking procedure in survey sampling (Deming & Stephan, 1940), which adjusts sampling weights to match known marginal totals. The covariate mean constraint can be written as

$$
\begin{equation*}
h_{nj}(\theta) = \frac{1}{n} \sum_{i=1}^n \{W_{ik} - E(W_{ik})\}/e(W_i; \theta) \leqslant Z_{\alpha_{1j}/2} \sigma_{nj}(\theta) / n^{1/2},
\end{equation*}$$

where $\sigma_{nj}(\theta)$ is the sample standard deviation of $\{W_{ik}-E(W_{ik})\}/e(W_i; \theta)$.

*Example* 4. Suppose that we are confident that higher values of $W_{ik}$ are associated with an increased probability of sample selection. For example, $W_{ik}$ could be years of education and we might know from comparisons with representative samples, such as the census, that better educated individuals are more likely to select into the sample, conditional on other selection variables, so that $\theta_j \geqslant 0$ a priori.

*Example* 5. Suppose we know that two variables $W_{ik}$ and $W_{ik^{{\rm{pr}}ime}}$ are uncorrelated in the population. The inverse-probability-weighted correlation between $W_{ik}$ and $W_{ik^{{\rm{pr}}ime}}$ should therefore be zero. For example, due to the independent assortment of chromosomes, biological sex and autosomal genetic variants should be independent in the population; however, Pirastu et al. (2021) demonstrated that there is significant correlation within the UK Biobank. This constraint can be formulated in several ways, for example by fixing the regression coefficient of $W_{ik}$ on $W_{ik^{{\rm{pr}}ime}}$ to be zero.

Examples 2, 3 and 5 are two-sided constraints such that we also want these inequalities to hold for $-h_{nj}(\theta)$.

*Remark* 3. In the population means setting, Miratrix et al. (2018) demonstrated how to place shape constraints on the weighted empirical distribution of the response. Their approach involves constructing the worst-case weighted distribution given the Aronow & Lee (2013) bounding assumptions; see the Supplementary Material. This results in a set that contains the oracle weighted distribution with probability approaching $1$. Provided we have a valid test, we can implement shape constraints within our framework without the need to characterize the worst-case weighted distribution. In the simplest case, we might want a variable to follow a known distribution in the population. For example, the distribution of IQ scores should be normal with mean 100 and standard deviation 15, which is a stronger constraint than Example 3. This could be formulated as a Kolmogorov–Smirnov test, and the relaxed constraint set could be constructed via the null distribution of that test.

## 4. Simulations

The aim of these simulations is to provide a brief assessment of the finite sample and limiting properties of the inference procedure described in § 2. For concreteness, we simulate the sensitivity analysis for selection bias described in § 3. Our parameter $\beta(\theta)$ and estimator $\beta_n(\theta)$ are both the coefficient of a weighted linear regression. In particular, a regression of $Y_i$ on $X_i$ for $(X_i, Y_i) \sim \mathcal{N}(0,I_2)$, where $I_2$ is the identity matrix. The weights take the form in (10) with variables $W_i = (X_i, Y_i)$.

We consider three distinct scenarios for the constraints. In the first scenario, we impose only sensitivity parameters $\Lambda_0^l = 0.11$, $\Lambda_0^u = 0.25$ and $\Lambda_1 = 3$. In the second scenario, we also impose a direction constraint $\theta_1 \geqslant 0$ as in Example 4. In the third scenario, we impose both the previous direction constraint, and set the response rate equal to $0.15$ as in Example 2. In each scenario, we use the discussion in Remark 2 to construct a two-sided 95$\%$ confidence interval for the identified set $I = [\nu^l, \nu^u]$, where $\nu^l = \inf\{\beta(\theta) \colon \theta \in \Theta\}$, $\nu^u = \sup\{\beta(\theta) \colon \theta \in \Theta\}$ and $\Theta$ takes the form in (14). The first and second scenarios have no sample constraints and so the confidence interval corresponds to that in (5). The third scenario employs the confidence interval proposed in (9) and Theorem 1.

Each scenario has distinct properties. In the first scenario, there are two solutions to the population optimization problems, thus violating Assumption 1. In the second scenario, the addition of a direction constraint rules out one of the two solutions and satisfies Assumption 1. In the third scenario, the introduction of a sample constraint necessitates the use of our relaxed confidence bound. In this scenario, we use our rule of thumb from Remark 1 to select $\alpha_1 = \alpha_2 = 0.025$ for both the upper and lower bounds of the two-sided confidence interval.

Table 1 summarizes the results and broadly aligns with our theoretical predictions. The first scenario violates Assumption 1 and the impact of this violation is substantial overcoverage of the confidence interval. Intuitively, this occurs because the sample solution will occur at, or near, the population solution that happens to minimize $\beta_n(\theta)$ in any given sample, which will result in a systematically wider confidence interval. The second scenario satisfies all assumptions for Proposition 2 and therefore converges to exact nominal coverage. The third scenario imposes a sample constraint and exhibits some overcoverage. This overcoverage can occur because our confidence bound in Theorem 1 sidesteps the covariance between the constraints $h_{nj}(\theta)$ and objective function $Q_n(\theta)$, instead imposing a worst-case intersection bound.

**Table 1.** Coverage frequency for the three scenarios over 5000 Monte Carlo replications

	Sample size						
Scenario	10	25	50	100	200	500	1000
1	0.972	0.992	0.995	0.997	0.998	0.996	0.995
2	0.936	0.974	0.981	0.983	0.979	0.966	0.947
3	0.953	0.985	0.991	0.991	0.987	0.986	0.979

In this simulation exercise, the weight model is comprised of two variables. The Supplementary Material contains an additional simulation exploring the computation time of our $\texttt{R}$ package $\texttt{selectioninterval}$ (R Development Core Team, 2023) as the number of variables in the weight model increases.

## 5. Applied example: effect of education on income

We consider an instrumental variable design looking at the effect of education on income in the UK Biobank cohort. Our instrument is exposure to an educational reform taking place in England in 1972. Our exposure is whether an individual remained in school until at least age 16, and our outcome is whether an individual earned more than £ 31 000 per year in 2006. We restrict our sample to individuals who turned 15 within 12 months of September 1972, and we control for sex and month-of-birth indicators. The unweighted estimate is 0.18 (95$\%$ confidence interval 0.08–0.28). An in-depth exposition of this design can be found in Davies et al. (2018) and the Supplementary Material.

We first apply the sensitivity analysis described in § 3.2 without auxiliary constraints, where the probability weights contain sex, years of education, income, age, days of physical activity per week, and an interaction term between education and income. We choose sensitivity parameters $\Lambda_0^l = 0.02$, $\Lambda_0^u = 0.25$ and $\Lambda_1 = 2$, so that the average individual in the sample has an odds of sample selection between $0.02$ and $0.25$, and each variable in the model can induce a marginal odds of sample selection between $0.5$ and $2$. Our sensitivity analysis suggests that the effect estimate lies in the interval $[-1.34, 0.94]$ (95$\%$ confidence interval $[-1.84, 1.29]$). This interval is completely uninformative as it spans the full range of possible estimates.

One explanation for this conservativeness is that this simple sensitivity analysis does not utilize all of the information available to us on the target population and the sample selection mechanism. The minimizing (maximizing) weights corresponding to this interval imply that the proportion of males in the population is 38.52$\%$ (46.6$\%$), and the proportion of households with a gross income greater than £ 31 000 is 95.84$\%$ (95.66$\%$), all of which are inconsistent with known characteristics of the U.K. population.

To address this incongruence, we consider four constraints that are typical of the information available to applied researchers using datasets such as the UK Biobank. The first constraint is the response rate of the UK Biobank (5.5$\%$), which is the proportion of individuals who entered the cohort after receiving an invitation. The second constraint is the proportion of males in the U.K. population within the UK Biobank age range of 40–69 (49.5$\%$). The third is the proportion of U.K. households earning more than £ 31 000 per year at the date of UK Biobank recruitment in 2006 (21$\%$). The fourth is the average age of individuals within our two-year age bracket (48.98). All statistics were obtained from publicly available records from the U.K.’s Office of National Statistics.

**Fig. 1.**
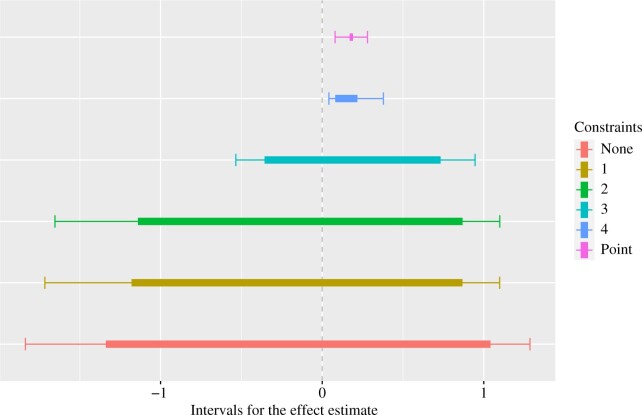
Estimated intervals (thick lines) and corresponding confidence intervals (thin lines) for effect estimates in the applied example. Point represents the unweighted point estimate. Each constraint is added sequentially. No constraint means that only the sensitivity parameters $\Lambda_0^l = 0.02$, $\Lambda_0^u = 0.25$ and $\Lambda_1=2$ are imposed. Constraint 1 sets the response rate equal to 5.5$\%$. Constraint 2 sets the proportion of males in the population to be 49.5$\%$. Constraint 3 sets the proportion of households earning more than £ 31 000 to be 21$\%$. Constraint 4 sets the average age of individuals to be 48.98 years.

Figure 1 shows the resulting estimated intervals (thicker lines) and their corresponding confidence intervals (thinner lines) where each constraint is added sequentially. The estimated intervals and confidence intervals correspond to those described in Remark 2. We can see that each additional constraint reduces the width of the interval, with constraints 3 and 4, the household income and age constraints, respectively, seemingly having the largest marginal impact. The top interval includes all constraints, and is quite informative for the desired effect, rejecting the null and suggesting an effect estimate in the range 0.08–0.22 (95$\%$ confidence interval 0.04–0.38). The unweighted estimate still lies within this interval, but our sensitivity analysis suggests some increased uncertainty in the range of effect estimates. These results also suggest that, despite the potential conservativeness of the confidence interval in Theorem 1, it can still produce informative bounds in practice.

## 6. Discussion

There has been some existing work on bootstrap inference for Rosenbaum-type sensitivity analyses (Zhao et al., 2019). This approach considers a fixed parameter space $\Theta$. It is unclear how to select the relaxation parameter $\epsilon_n(\theta)$ in a bootstrap analogue of our method under estimated constraints. Simple approaches, such as constructing $\Theta_n^r$ via asymptotic approximations and then bootstrapping the distribution of $\nu_n^r$, are plausible, but their statistical properties remain to be explored.

In some instances, including our selection bias application in § 3, the target of inference is $Q(\theta^*)$, where $\theta^*$ is some true parameter lying within $\Theta$, rather than $\nu$. Suppose that $\Theta$ is known and that we have a two-sided identified set $[\inf_{\theta \in \Theta} Q(\theta), \sup_{\theta \in \Theta} Q(\theta)]$ as in Remark 2; then if $Q(\theta^*)$ lies near the boundary of this set, and the set has positive width, the noncoverage probability of the corresponding confidence interval is effectively one-sided in the limit. A naive two-sided confidence interval constructed around the identified set may be too conservative. Imbens & Manski (2004) discussed approaches for maintaining uniform coverage of $Q(\theta^*)$. The central limit theorem established by Shapiro (1991) for known $\Theta$ is amenable to their framework, although, to our knowledge, has not been formally used in this setting; extending this result to sample-constrained problems would be a valuable contribution. Stoye (2009) further extended the work of Imbens & Manski (2004) by developing confidence intervals that exhibit uniform coverage for $Q(\theta^*)$, without relying on assumed superefficiency of the estimated interval width.

A final consideration is the computational burden of our approach. Our general inference procedure in § 2 relies on the optimization problems in (8) being solvable, but the computational complexity of these problems will vary, depending on the application. Our $\texttt{R}$ package $\texttt{selectioninterval}$ relies on out-of-the-box global and local optimization algorithms. There are no theoretical guarantees of convergence to the global optimum; however, we have not observed a failure of convergence in our simulations.

## Supplementary Material

asac042_Supplementary_Data

## Supplementary material

The Supplementary Material includes proofs for the results in § 2, further details for the applied example in § 5 and an extension of Aronow & Lee (2013). An $\texttt{R}$ package to implement the sensitivity analysis described in § 3 is available at https://github.com/matttudball/selectioninterval.
