# Supplementary material for: Sample-constrained partial identification with application to selection bias
Source: Biometrika. 2022 Jul 25;110(2):485–98. doi: 10.1093/biomet/asac042 (PMC10183833; doi:10.1093/biomet/asac042)
Supplement: asac042_Supplementary_Data [file asac042_supplementary_data.pdf]

Biometrika (2020), **103**, 1, *p.* 1
Printed in Great Britain Advance Access publication on 31 July 2018

Supplementary material for "Sample-constrained partial identification with application to selection bias"

BY MATTHEW J. TUDBALL
MRC Integrative Epidemiology Unit, University of Bristol, Oakfield Grove, Bristol, BS8 2BN, U.K.
matt.tudball@bristol.ac.uk

RACHAEL A. HUGHES, KATE TILLING
MRC Integrative Epidemiology Unit, University of Bristol, Oakfield Grove, Bristol, BS8 2BN, U.K.
rachael.hughes@bristol.ac.uk kate.tilling@bristol.ac.uk

JACK BOWDEN
College of Medicine and Health, University of Exeter, Heavitree Road, Exeter, EX1 2LU, U.K.
j.bowden2@exeter.ac.uk

QINGYUAN ZHAO
Department of Pure Mathematics and Mathematical Statistics, University of Cambridge, Wilberforce Road, Cambridge CB3 0WB, U.K.
qyzhao@statslab.cam.ac.uk

1. FURTHER DETAILS FOR THE APPLIED EXAMPLE

1·1. *Description of the design*

We implement an instrumental variable design for the effect of education on income in the UK Biobank cohort (Davies et al., 2018). The proposed instrument is based on a September 1972 education reform in England which raised the school leaving age from 15 to 16. Individuals who turned 15 just prior to the implementation of this reform were allowed to leave school, while individuals who turned 15 just after were required to remain in school until they were 16. This created a sharp discontinuity in the policies that the two groups were exposed to. Under the assumption that individuals on either side of the age threshold are otherwise identical, we can use this policy reform as an instrumental variable.

1·2. *Varying the sensitivity parameters*

It is important to report a few choices for the sensitivity parameters to understand which parameters are driving the width of the interval. Selecting $\Lambda_1 < 1.75$ results in an empty constraint set, indicating that there are no parameters which satisfy all of the auxiliary information constraints provided. The response rate constraint of Example 2 appears to be more informative when the interval $(\Lambda_0^l, \Lambda_0^u)$ is wider, which is expected. For all choices of parameters we consider, the constraints are informative and recover an interval that rejects the null.

© 2017 Biometrika Trust

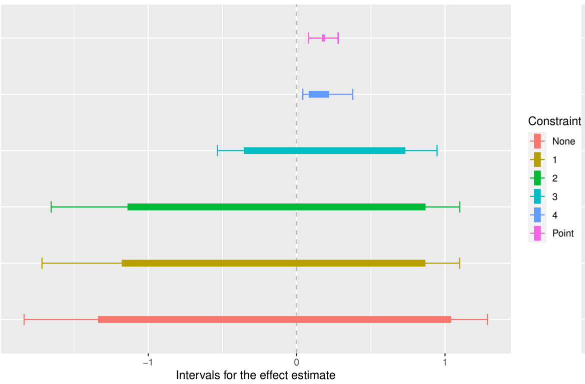

(a) $\Lambda_0^l = 0.02, \Lambda_0^u = 0.2, \Lambda_1 = 2$

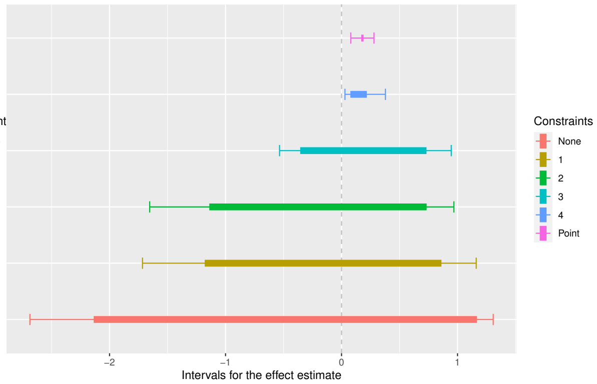

(b) $\Lambda_0^l = 0.01, \Lambda_0^u = 0.5, \Lambda_1 = 2$

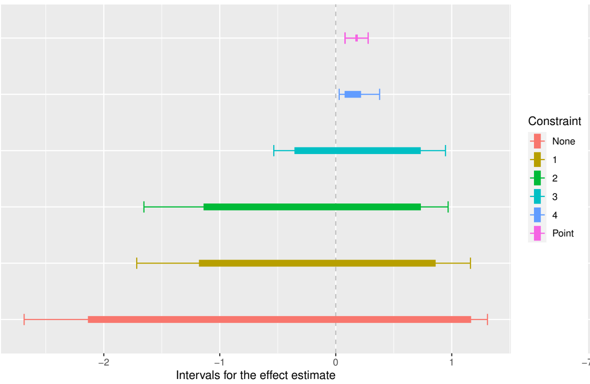

(c) $\Lambda_0^l = 0.02, \Lambda_0^u = 0.2, \Lambda_1 = 2.5$

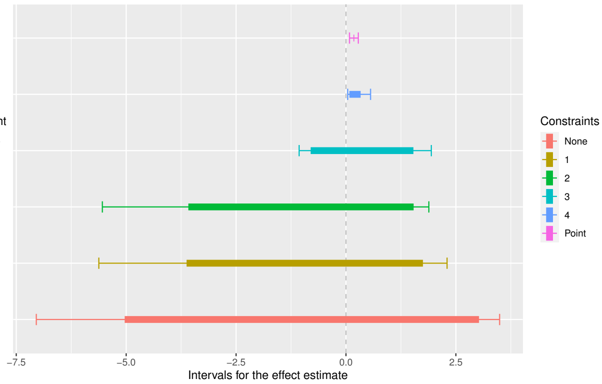

(d) $\Lambda_0^l = 0.01, \Lambda_0^u = 0.5, \Lambda_1 = 2.5$

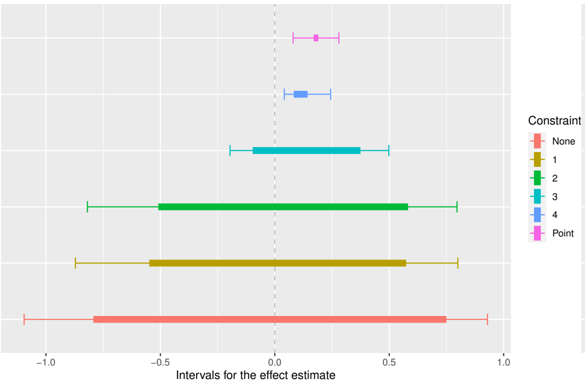

(e) $\Lambda_0^l = 0.02, \Lambda_0^u = 0.2, \Lambda_1 = 1.75$

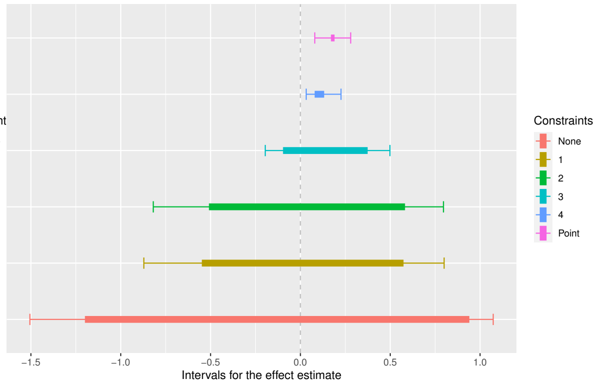

(f) $\Lambda_0^l = 0.01, \Lambda_0^u = 0.5, \Lambda_1 = 1.75$

Fig. 1: This figure presents several choices of sensitivity parameters for the applied example described in Section 5.

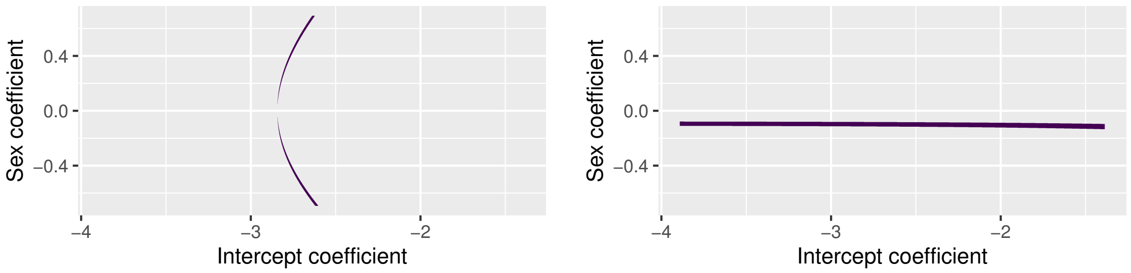

(a) Response rate constraint

(b) Covariate mean constraint

Fig. 2: This figure plots the feasible region (purple region) for a simple selection model with one variable and an intercept. Panel (a) sets the response rate to be 0.055 and panel (b) sets the population mean of male sex to be 0.495.

1·3. Visualizing the feasible region

It is also illustrative to plot the feasible region for a couple of simple examples. Suppose that W_i consists only of the sex variable. We select sensitivity parameters $(\Lambda_0^l, \Lambda_0^u, \Lambda_1) = (0.02, 0.2, 2)$ as usual and consider two constraints: setting the response rate to be 0.055 and setting the population mean of male sex to be 0.495.

Figure 2 plots the two feasible regions. The feasible regions are both small in comparison to the space implied by the sensitivity parameters. Imposing both constraints simultaneously will result in a non-empty feasible region.

1·4. Implied selection probabilities within covariate strata

We can check the implied probabilities of sample selection within different covariate strata. Strata for which the implied probabilities are extreme, or inconsistent with known patterns of sample selection, could be addressed by introducing additional constraints. To illustrate this idea, Tables 1 and 2 show the implied probabilities within strata of sex and educational attainment of the sensitivity analysis in Section 5. Table 1 shows the probabilities with only the response rate constraint and Table 2 shows the probabilities with all constraints.

The probabilities in Table 1 exhibit sample selection patterns that are inconsistent with known characteristics of UK Biobank (Fry et al., 2017). In particular, at both the lower and upper bounds, better educated individuals are less likely to select into the sample. At the lower bound, men are more likely to select into the sample than women.

The probabilities in Table 2 are more consistent with UK Biobank selection patterns. At the lower bound, better educated individuals are now more likely to select into the sample. Women are more likely to select into the sample than men across most strata of educational attainment. Despite this, the selection pattern for education is still contrary to our expectations at the upper bound. This could indicate that a constraint on average educational attainment would tighten the upper bound. Alternatively, we could constrain the coefficient for educational attainment to be non-negative in the weight model.

2. COMPUTATION TIME OF SELECTION BIAS METHOD

This simulation illustrates the computation time of our R package `selectioninterval` as the number of weight model increases. We replicate the simulation set-up in Section 4. To reiterate, our parameter is the regression coefficient of Y_i on X_i for $(X_i, Y_i) \sim \mathcal{N}(0, I_2)$, $i =$

Table 1: Implied probabilities across sex and education strata, response rate constraint

Age finished school	Probabilities at lower bound		Probabilities at upper bound	
	Female	Male	Female	Male
14	0.59	0.72	0.33	0.17
15	0.43	0.60	0.27	0.12
16	0.40	0.56	0.35	0.17
17	0.28	0.43	0.31	0.13
18	0.22	0.33	0.28	0.11
19	0.21	0.30	0.22	0.08
20	0.14	0.30	0.19	0.07
21	0.09	0.17	0.17	0.06
22	0.10	0.16	0.11	0.04

Table 2: Implied probabilities across sex and education strata, all constraints

Age finished school	Probabilities at lower bound		Probabilities at upper bound	
	Female	Male	Female	Male
14	0.07	0.06	0.20	0.17
15	0.10	0.09	0.20	0.17
16	0.13	0.12	0.21	0.18
17	0.16	0.17	0.21	0.17
18	0.19	0.20	0.19	0.16
19	0.20	0.22	0.15	0.13
20	0.24	0.22	0.14	0.10
21	0.29	0.28	0.13	0.10
22	0.27	0.31	0.09	0.08

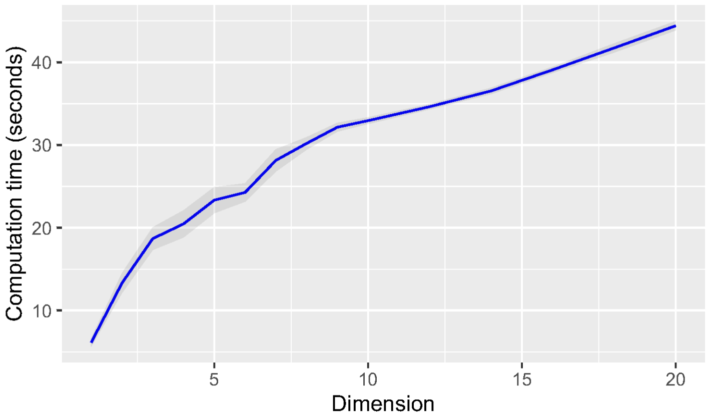

Fig. 3: This figure plots the computation time in seconds of our R package for a sample size of 200. The dimension d is varied between 1 and 20.

$1, \ldots, 200$. The variables in the weight model are $W_i = (X_i, Y_i, Z_i^d)$, where $Z_i^d \sim \mathcal{N}(0, I_d)$. Figure 3 shows the single-core computation time in seconds of the lower and upper bounds as the dimension d is varied from 1 to 20. For this simulation, computational complexity appears to be sub-linear.

3. SUFFICIENT CONDITIONS FOR ASSUMPTION 6

Assumption 1. For sufficiently large n, $\Theta_n^r \subseteq B$ with probability one.

Assumption 2. For all $j = 1, \ldots, J$, $h_{nj}(\theta)$ converges to $h_j(\theta)$ and $\epsilon_{nj}(\theta)$ converges to 0 with probability one as $n \to \infty$ uniformly on B.

Assumption 3. For any $\theta \in \Theta$, let $\mathcal{A}(\theta) = \{j \colon h_j(\theta) = 0\}$ be the indices of active constraints, which could be empty. We assume that the gradient vectors $\nabla h_j(\vartheta)$, $j \in \mathcal{A}(\vartheta)$, are linearly independent.

Under these assumptions, Assumption 6 is an immediate corollary of Lemma 3.

4. EXTENSION OF ARONOW & LEE (2013)

4·1. *Extension to ratio estimators*

Another sensitivity analysis that can be made more informative through the addition of auxiliary constraints is one proposed in Aronow & Lee (2013). This non-parametric sensitivity analysis computes bounds on an inverse probability weighted sample mean under the assumption that each weight is bounded between two known constants. To start, we provide a slight generalization of this sensitivity analysis to ratio estimators. Let the estimator be given by

$$\beta_n = \left(\sum_{i=1}^{n} f(T_i)/e(W_i)\right) \Big/ \left(\sum_{i=1}^{n} g(T_i)/e(W_i)\right) \tag{1}$$

where $T_i \in \mathcal{T} \subseteq \mathbb{R}^M$ and $f, g \colon \mathbb{R}^M \to \mathbb{R}$. We assume that each $e(W_i)$ is unknown (and some W_i may be unmeasured) and lies between two user-specified constants $1 \le a \le e(W_i) \le b < \infty$.

To apply our theoretical results in Section 2, we need a set Θ of fixed dimension. A simple assumption is that $\mathcal{T}$ is discrete and finite, $\mathcal{T} = \{t_k \colon k = 1, 2, \ldots, K\}$. Under this assumption, we can define $\beta_n(\theta)$ equal to

$$\beta_n(\theta) = \left(\sum_{k=1}^{K} \theta_k f(t_k) p_n(t_k)\right) \Big/ \left(\sum_{k=1}^{K} \theta_k g(t_k) p_n(t_k)\right)$$
$$\beta(\theta) = \left(\sum_{k=1}^{K} \theta_k f(t_k) p(t_k)\right) \Big/ \left(\sum_{k=1}^{K} \theta_k g(t_k) p(t_k)\right)$$

where $\theta_k = E[1/e(W_i) \mid T_i = t_k, S_i = 1]$ and $p_n(\cdot), p(\cdot)$ are (respectively) the sample and population probability measures. This results in $\Theta = [1/b, 1/a]^K$. From here, the infimum takes the usual form,

$$\nu_n = \inf\{\beta_n(\theta) \colon \theta \in \Theta\}, \quad \nu = \inf\{\beta(\theta) \colon \theta \in \Theta\}.$$

It remains to identify assumptions such that the conditions in Section 2 are satisfied.

Assumption 4. For all $\theta \in \Theta$, the denominators of $\beta(\theta)$ and $\beta_n(\theta)$ are non-zero with probability one.

Assumption 5. For all $t_k \in \mathcal{T}$, $f(t_k)/g(t_k) \neq \nu$.

Assumption 4 simply ensures that $\beta_n(\theta)$ and $\beta(\theta)$ are well-defined over Θ. Assumption 5 is more subtle but will be needed to ensure a unique solution. If this assumption violated for some k, then the infimum is identical for all values of θ_k, meaning that the solution is not unique. An example of a function violating this condition is the following:

$$\beta(\theta) = \frac{-\theta_1 - 7\theta_2 - 10\theta_3}{\theta_1 + \theta_2 + \theta_3}, \quad \Theta = [1, 2]^3$$

In this example, $\vartheta = (1,1,2)$ and $\vartheta = (1,2,2)$ are both minimizers over Θ. The minimum value is -7 but $f(t_2)/g(t_2) = -7$ as well, which violates the condition. These assumptions jointly imply that ν has a unique minimizer. We begin with a technical lemma.

LEMMA 1. *$\nu_n = Q_n(\vartheta_n)$ is a global minimum over Θ if and only if, for all $k = 1, ..., K$, $q_k f(t_k) \leq \nu\, q_k\, g(t_k)$, where $q_k = \vartheta_k - (1/a + 1/b - \vartheta_k)$.*

This leads to our main proposition.

PROPOSITION 1. *Under Assumptions 4 and 5, the set $\{\theta \in \Theta\colon \beta(\theta) = \nu\}$ is a singleton and, for n sufficiently large, the set $\{\theta \in \Theta\colon \beta_n(\theta) = \nu_n\}$ is a singleton with probability one.*

In fact, Proposition 1 provides an explicit form for the sample minimizer,

$$\vartheta_{nk} = \begin{cases} 1/b & \text{if } f(t_k)/g(t_k) \geq \nu_n \\ 1/a & \text{if } f(t_k)/g(t_k) < \nu_n \end{cases}, \tag{2}$$

and the population minimizer takes a similar form but with ν_n replaced with ν. Both Proposition 1 of Aronow & Lee (2013) and Section 4.4 of Zhao et al. (2019) propose equivalent algorithms for computing the optimizing weights in the population means setting. Proposition 1 shows that we can generalize this algorithm to ratio estimators. In short, we can order $f(t_k)/g(t_k)$ from smallest to largest and evaluate $\beta_n(\theta)$ by enumerating over the weight at which $1/b$ changes to $1/a$, which has computational complexity $O(n)$.

Proposition 1 shows that Assumption 1 is satisfied for the generalized Aronow & Lee (2013) estimator under relatively weak conditions. Furthermore, Assumptions 2, 4 and 3 are satisfied under Assumption 4 and the assumption that $\mathcal{T}$ is finite. We therefore have that Proposition 2 is satisfied under these same relatively weak conditions.

4·2. *Auxiliary information constraints*

The constraints described in Examples 2 and 3 can be applied to this estimator. Let $c_n = Z_{\alpha_{1j}/2}\, n^{-1/2}$, then the response rate constraint can be formulated as

$$h_{nj}(\theta) = \left(1 + c_n^2\right)\left\{\sum_{k=1}^{K}(\theta_k - 1/r)p_n(t_k)\right\}^2 - c_n^2 \sum_{k=1}^{K}(\theta_k - 1/r)^2 p_n(t_k) \leq 0. \tag{3}$$

Suppose $w_k \in \mathbb{R}$ is an element of t_k, then the covariate mean constraint can be formulated as

$$h_{nj}(\theta) = \left(1 + c_n^2\right)\left\{\sum_{k=1}^{K}\theta_k(w_k - \bar{w})p_n(t_k)\right\}^2 - c_n^2 \sum_{k=1}^{K}\theta_k^2(w_k - \bar{w})^2 p_n(t_k) \leq 0. \tag{4}$$

Both of these constraints are quadratic in θ and can therefore be solved by existing algorithms for quadratically-constrained linear programs. Example 4 cannot be extended to this setting because it is tied to a parametric model. Example 5 can be extended to this setting in principle, but the resulting optimization problem is intractable.

Uniqueness of ϑ over Θ is needed to invoke Theorem 1. The population minimization problem over Θ is a linearly-constrained linear fractional programming problem. For example, the population response rate constraint is

$$h_j(\theta) = \sum_{k=1}^{K}(\theta_k - 1/r)p(t_k),$$

which is linear in θ. Since the level sets of $\beta(\theta)$ are also linear, to establish uniqueness of ϑ it suffices to assume (in addition to Assumptions 4 and 5) that the coefficient vectors of $h_j(\theta)$ and the level sets of $\beta(\theta)$ over Θ are not parallel.

5. Technical details

Proof of Lemma 1

Proof. Consider the population problem. We will prove the 'if' statement since the 'only if' statement follows from some simple algebra. We will begin by noting that, since ν is the solution to a linear fractional programming problem and since Θ is a compact, convex polyhedron, the maximizing weight vector ϑ will lie at a vertex. In other words, $\vartheta \in \{1/b,\, 1/a\}^K$. Take an arbitrary weight vector $\theta \in \{1/b,\, 1/a\}^K$. Suppose there are $1 < m \leq K$ elements of θ which differ from ϑ. Without loss of generality, suppose these are the first m elements. Then we can write

$$
\begin{aligned}
\beta(w) &= \frac{\sum_{k=1}^{K} \theta_k f(t_k) p(t_k)}{\sum_{k=1}^{K} \theta_k g(t_k) p(t_k)} \\
&= \frac{\sum_{k=1}^{K} \vartheta_k f(t_k) p(t_k) - \sum_{k=1}^{m} q_k f(t_k) p(t_k)}{\sum_{k=1}^{K} \vartheta_j g(t_k) p(t_k) - \sum_{k=1}^{m} q_k g(t_k) p(t_k)} \\
&\geq \frac{\nu \sum_{k=1}^{K} \vartheta_k g(t_k) p(t_k) - \nu \sum_{k=1}^{m} q_k g(t_k) p(t_k)}{\sum_{k=1}^{K} \vartheta_k g(t_k) p(t_k) - \sum_{k=1}^{m} q_k g(t_k) p(t_k)} \\
&= \nu \, \frac{\sum_{k=1}^{K} \vartheta_k g(t_k) p(t_k) - \sum_{k=1}^{m} q_k g(t_k) p(t_k)}{\sum_{k=1}^{K} \vartheta_k g(t_k) p(t_k) - \sum_{k=1}^{m} q_k g(t_k) p(t_k)} \\
&= \nu
\end{aligned}
$$

The same holds with probability one for the sample problem by replacing $p(t_k)$ with $p_n(t_k)$. □

Proof of Proposition 1

Proof. Consider the population problem. Suppose that there are two global minima, $\nu_1 = \beta(\vartheta_1)$ and $\nu_2 = \beta(\vartheta_2)$, such that $\nu_1 = \nu_2 = \nu$ and $\vartheta_1 \neq \vartheta_2$. Since ν_1 and ν_2 are both global minima then, by Lemma 1, for all $k = 1, \ldots, K$,

$$
\begin{aligned}
q_k\, f(t_k) &\leq \nu_1\, q_k\, g(t_k) \\
q_k\, f(t_k) &\leq \nu_2\, q_k\, g(t_k)
\end{aligned}
\tag{5}
$$

Without loss of generality, we assume that ϑ_1 and ϑ_2 differ by the first m elements. Then,

$$
\begin{aligned}
\nu = \nu_1 &= \frac{\sum_{k=1}^{K} \vartheta_{1j} f(t_k) p(t_k)}{\sum_{k=1}^{K} \vartheta_{1k} g(t_k) p(t_k)} \\
&= \frac{\sum_{k=1}^{K} \vartheta_{2k} f(t_k) p(t_k)}{\sum_{k=1}^{K} \vartheta_{2k} g(t_k) p(t_k)} \\
&= \frac{\sum_{k=1}^{K} \vartheta_{1k} f(t_k) p(t_k) - \sum_{k=1}^{K} q_k f(t_k) p(t_k)}{\sum_{k=1}^{K} \vartheta_{1k} g(t_k) p(t_k) - \sum_{k=1}^{m} q_k g(t_k) p(t_k)}
\end{aligned}
$$

where $q_k = \vartheta_{1k} - (1/a + 1/b - \vartheta_{1k})$. Rearranging, we obtain,

$$\sum_{k=1}^{m} q_k \, f(t_k) \, p(t_k) \;=\; \nu \sum_{k=1}^{m} q_k \, g(t_k) \, p(t_k)$$

However, (5) implies that this equality will only hold if, for all $k = 1, ..., m$, $f(t_k)/g(t_k) = \nu$, which cannot be true by Assumption 1. Therefore, by contradiction, the set $\{\theta \in \Theta \colon \beta(\theta) = \nu\}$ must be a singleton. The same holds with probability one for the sample problem by replacing $p(t_k)$ with $p_n(t_k)$. □

Proof of Proposition 3

Proof.

$$\begin{aligned} |\sigma_n^2(\vartheta_n) - \sigma^2(\vartheta)| &= |\sigma_n^2(\vartheta_n) - \sigma^2(\vartheta_n) + \sigma^2(\vartheta_n) - \sigma^2(\vartheta)| \\ &\le |\sigma_n^2(\vartheta_n) - \sigma^2(\vartheta_n)| \,+\, |\sigma^2(\vartheta_n) - \sigma^2(\vartheta)| \\ &\le \sup_{\theta \in \Theta} |\sigma_n^2(\theta) - \sigma^2(\theta)| \,+\, |\sigma^2(\vartheta_n) - \sigma^2(\vartheta)| \\ &\to 0 \text{ with probability one,} \end{aligned}$$

where the last inequality holds by the uniform strong consistency and the second term on the last line goes to zero with probability one since $\vartheta_n \to \vartheta$ with probability one by Proposition 1 and $\sigma^2(\theta) \in C(S)$. □

Before proving Theorem 1, we begin with some notation and preliminary lemmas. Denote the confidence bound for a particular θ and α as

$$C_n(\theta, \alpha) = Q_n(\theta) - Z_\alpha \, \sigma_n(\theta) \, n^{-1/2}$$

and the sample minimum over Θ_n^r at α_2 as

$$\zeta_n^r \in \arg\min\{C_n(\theta, \alpha_2) \colon \theta \in \Theta_n^r\}.$$

Recall that $\vartheta_n^r \in \arg\min\{Q_n(\theta) \colon \theta \in \Theta^r\}$ and $\vartheta = \arg\min\{Q(\theta) \colon \theta \in \Theta\}$, which is assumed to be unique. These quantities can be ordered deterministically as

$$C_n(\zeta_n^r, \alpha) \;\le\; C_n(\vartheta_n^r, \alpha) \;\le\; Q_n(\vartheta_n^r) \;\le\; Q_n(\zeta_n^r).$$

The first lemma provides a lower bound for the coverage probability of $C_n(\vartheta_n^r, \alpha_2)$.

LEMMA 2. *Let $\delta_n = Z_{\alpha_2} \, \epsilon \, n^{-1/2}$ be a deterministic sequence where $\epsilon > 0$ is any positive constant, then*

$$\mathrm{pr}\Big(C_n(\vartheta_n^r, \alpha_2) \;\le\; \nu\Big) \;\ge\; \mathrm{pr}\Big(C_n(\zeta_n^r, \alpha_2) \;\le\; \nu - \delta_n\Big) \;+\; \mathrm{pr}\Big(\big|\sigma_n(\zeta_n^r) \;-\; \sigma_n(\vartheta_n^r)\big| \;\le\; \epsilon\Big) \;-\; 1.$$

Proof.

$$
\begin{aligned}
&\mathrm{pr}\Big(C_n(\vartheta^r_n, \alpha_2) \,\le\, \nu \Big) \\
&= \mathrm{pr}\Big(Q_n(\vartheta^r_n) \,-\, Z_{\alpha_2}\, \sigma_n(\vartheta^r_n)\, n^{-1/2} \,\le\, \nu \Big) \\
&= \mathrm{pr}\Big(Q_n(\zeta^r_n) \,-\, Z_{\alpha_2} \sigma_n(\zeta^r_n) n^{-1/2} \,+\, \big\{ (Q_n(\vartheta^r_n) - Q_n(\zeta^r_n) \big\} \,+\, Z_{\alpha_2} \big\{ \sigma_n(\zeta^r_n) \,-\, \sigma_n(\vartheta^r_n) \big\} n^{-1/2} \,\le\, \nu \Big) \\
&\ge \mathrm{pr}\Big(Q_n(\zeta^r_n) \,-\, Z_{\alpha_2} \sigma_n(\zeta^r_n) n^{-1/2} \,+\, Z_{\alpha_2} \big\{ \sigma_n(\zeta^r_n) \,-\, \sigma_n(\vartheta^r_n) \big\} n^{-1/2} \,\le\, \nu \Big) \\
&\ge \mathrm{pr}\Big(Q_n(\zeta^r_n) \,-\, Z_{\alpha_2} \sigma_n(\zeta^r_n) n^{-1/2} \,+\, \delta_n \,\le\, \nu,\, Z_{\alpha_2} \big| \sigma_n(\zeta^r_n) \,-\, \sigma_n(\vartheta^r_n) \big| n^{-1/2} \,\le\, \delta_n \Big) \\
&\ge \mathrm{pr}\Big(C_n(\zeta^r_n, \alpha_2) \,\le\, \nu - \delta_n \Big) \,+\, \mathrm{pr}\Big(Z_{\alpha_2} \big| \sigma_n(\zeta^r_n) \,-\, \sigma_n(\vartheta^r_n) \big| n^{-1/2} \,\le\, \delta_n \Big) \,-\, 1 \\
&= \mathrm{pr}\Big(C_n(\zeta^r_n, \alpha_2) \,\le\, \nu - \delta_n \Big) \,+\, \mathrm{pr}\Big(\big| \sigma_n(\zeta^r_n) \,-\, \sigma_n(\vartheta^r_n) \big| \,\le\, \epsilon \Big) \,-\, 1.
\end{aligned}
$$

LEMMA 3. *Suppose a sequence of functions $\tilde{Q}_n \colon \mathbb{R}^p \to \mathbb{R}$ is in $C(B)$ and converges to $Q(\theta)$ with probability one as $n \to \infty$ uniformly on B. Furthermore, let $\tilde{\nu}_n = \inf\{\tilde{Q}_n(\theta) \colon \Theta^r_n\}$ and $\tilde{\vartheta}_n \in \{\theta \colon \tilde{Q}_n(\theta) = \tilde{\nu}_n\}$. Then, under Assumptions 1, 2 and 1 - 3, $\tilde{\nu}_n \to \nu$ and $\tilde{\vartheta}_n \to \vartheta$ with probability one as $n \to \infty$.*

Proof. The proof of this lemma combines Theorem 5.3 and Theorem 5.5 in Shapiro et al. (2009). Theorem 5.3 establishes consistency of optimal values and solutions when Θ is known. Theorem 5.5 generalizes this result to an estimated constraint set, in our case Θ^r_n.

In particular, Theorem 5.5 requires that the following two conditions are satisfied:

a) If $\theta_n \in \Theta^r_n$ and θ_n converges with probability one to a point θ^*, then $\theta^* \in \Theta$.
b) There exists a sequence $\theta_n \in \Theta^r_n$ such that θ_n converges to ϑ with probability one.

We begin with the proof of condition (a). Suppose $\theta^* \notin \Theta$. Then there exists some $j = 1, \ldots, J$ such that $h_j(\theta^*) \ge \delta$, where $\delta > 0$ is some constant. By the triangle inequality,

$$|h_{jn}(\theta_n) - h_j(\theta^*)| \le |h_{jn}(\theta_n) - h_{jn}(\theta^*)| + |h_{jn}(\theta^*) - h_j(\theta^*)|.$$

The first term converges to zero with probability one because θ_n converges to θ^* and $h_{jn}(\theta) \in C(B)$, both with probability one. The second term also converges to zero with probability one by Assumption 2 because $h_{jn}(\theta) \to h_j(\theta)$ uniformly on B with probability one. This means that for all $\epsilon > 0$ there exists an $n \ge n_\epsilon$ such that

$$|h_{jn}(\theta_n) - h_j(\theta^*)| < \epsilon.$$

However, since $h_j(\theta^*) \ge \delta$ and $\theta_n \in \Theta^r_n$, such that $h_{jn}(\theta_n) \le \epsilon_{jn}(\theta_n)$, this is equivalent to

$$h_j(\theta^*) - h_{jn}(\theta_n) < \epsilon$$

whenever $\delta > \epsilon_{jn}(\theta_n)$. Without loss of generality, we can set $\epsilon = \delta - \epsilon_{jn}(\theta) > 0$. From here, we can rearrange,

$$h_{jn}(\theta_n) > h_j(\theta^*) - \epsilon \geq \delta - \epsilon \geq \epsilon_{jn}(\theta_n),$$

which means that $\theta_n \notin \Theta_n^r$, which is a contradiction. Therefore, it must be that $\theta^* \in \Theta$.

Condition (b) follows from the constraint qualification imposed in Assumption 3 and the discussion in (Shapiro et al., 2009, p. 161-162). □

Proof of Theorem 1

Proof. We begin by invoking Lemma 2, which states that

$$\text{pr}\Big(C_n(\vartheta_n^r, \alpha_2) \leq \nu\Big) \geq \text{pr}\Big(C_n(\zeta_n^r, \alpha_2) \leq \nu - \delta_n\Big) + \text{pr}\Big(\big|\sigma_n(\zeta_n^r) - \sigma_n(\vartheta_n^r)\big| \leq \epsilon\Big) - 1,$$

where $\delta_n = Z_{\alpha_2}\, \epsilon\, n^{-1/2}$ and $\epsilon > 0$ is any positive constant. We claim that

$$\lim_{n\to\infty} \text{pr}\Big(\big|\sigma_n(\zeta_n^r) - \sigma_n(\vartheta_n^r)\big| \leq \epsilon\Big) = 1.$$

This follows from Proposition 3 and Assumption 6. A sufficient condition for satisfying this assumption is that $\vartheta_n^r \to \vartheta$ and $\zeta_n^r \to \vartheta$ with probability one. This follows from Lemma 3 since $Q_n(\theta)$ and $C_n(\theta, \alpha)$ both converge to $Q(\theta)$ with probability one uniformly on B by Assumption 3. Therefore, we have that

$$\begin{aligned}
&\lim_{n\to\infty} \text{pr}\Big(C_n(\vartheta_n^r, \alpha_2) \leq \nu\Big) \\
&\geq \lim_{n\to\infty} \text{pr}\Big(C_n(\zeta_n^r, \alpha_2) \leq \nu - \delta_n\Big) \\
&\geq \lim_{n\to\infty} \text{pr}\Big(C_n(\zeta_n^r, \alpha_2) \leq \nu - \delta_n,\ \Theta \subseteq \Theta_n^r\Big) \\
&\geq \lim_{n\to\infty} \text{pr}\Big(C_n(\vartheta_n, \alpha_2) \leq \nu - \delta_n,\ \Theta \subseteq \Theta_n^r\Big) \\
&= \lim_{n\to\infty} \Big\{\text{pr}\Big(Q_n(\vartheta_n) - Z_{\alpha_2}\sigma_n(\vartheta_n) n^{-1/2} \leq \nu - \delta_n\Big) - \text{pr}\Big(C_n(\vartheta_n, \alpha_2) \leq \nu - \delta_n,\ \Theta \nsubseteq \Theta_n^r\Big)\Big\} \\
&\geq \lim_{n\to\infty} \Big\{\text{pr}\Big(n^{1/2}\,\frac{(Q_n(\vartheta_n) - \nu)}{\sigma(\vartheta)}\,\frac{\sigma(\vartheta)}{\sigma_n(\vartheta_n)} \leq Z_{\alpha_2}(1-\epsilon)\Big) - \text{pr}\Big(\Theta \nsubseteq \Theta_n^r\Big)\Big\} \\
&\geq \Phi(Z_{\alpha_2}(1-\epsilon)) - \alpha_1
\end{aligned}$$

where the last inequality follows by Slutsky's theorem, Proposition 3 and Proposition 2. Since $\epsilon > 0$ is an arbitrarily small constant, this lower bound can be set arbitrarily close to $1 - \alpha_2 - \alpha_1$. □

References

Aronow, P. M. & Lee, D. K. (2013). Interval estimation of population means under unknown but bounded probabilities of sample selection. *Biometrika* **100**, 235–240.

Davies, N. M., Dickson, M., Davey Smith, G., Van Den Berg, G. J. & Windmeijer, F. (2018). The causal effects of education on health outcomes in the UK Biobank. *Nature Human Behaviour* **2**, 117–125.

Fry, A., Littlejohns, T. J., Sudlow, C., Doherty, N., Adamska, L., Sprosen, T., Collins, R. & Allen, N. E. (2017). Comparison of sociodemographic and health-related characteristics of UK Biobank participants with those of the general population. *American Journal of Epidemiology* **186**, 1026–1034.

Shapiro, A., Dentcheva, D. & Ruszczyński, A. (2009). *Lectures on Stochastic Programming: Modeling and Theory*. Society for Industrial and Applied Mathematics and the Mathematical Programming Society.

Zhao, Q., Small, D. S. & Bhattacharya, B. B. (2019). Sensitivity analysis for inverse probability weighting estimators via the percentile bootstrap. *Journal of the Royal Statistical Society: Series B* **81**, 1–27.

[*Received 2 January 2017. Editorial decision on 1 April 2017*]
